# Cardiac Peroxisome Proliferator-Activated Receptor-γ Expression is Modulated by Oxidative Stress in Acutely Infrasound-Exposed Cardiomyocytes

**DOI:** 10.1007/s12012-013-9211-5

**Published:** 2013-05-01

**Authors:** Zhaohui Pei, Rongsen Meng, Zhiqiang Zhuang, Yiqiao Zhao, Fangpeng Liu, Miao-Zhang Zhu, Ruiman Li

**Affiliations:** 1 Department of Cardiology, The Third Hospital of Nanchang, Nanchang, 330009 Jiangxi China; 2 Department of Cardiology, The Second People’s Hospital of Guangdong Province, Guangzhou, China; 3 Department of Rehabilitation Medicine, The Second Affiliated Hospital of Sun Yat-Sen University, Guangzhou, Guangdong Province China; 4 Department of Cardiology, The Third Affiliated Hospital of Nanchang University, Nanchang, Jiangxi Province China; 5 Department of Physiology, The Fourth Military Medical University, Xi’an, China; 6 Department of Gynecology and Obstetrics, The First Affiliated Hospital of Jinan University, Guangzhou, China

**Keywords:** Infrasound exposure, Cardiomyocyte, Oxidative damage, PPAR-γ, Cytotoxicity

## Abstract

The aim of the present study was to examine the effects of acute infrasound exposure on oxidative damage and investigate the underlying mechanisms in rat cardiomyocytes. Neonatal rat cardiomyocytes were cultured and exposed to infrasound for several days. In the study, the expression of CAT, GPx, SOD1, and SOD2 and their activities in rat cardiomyocytes in infrasound exposure groups were significantly decreased compared to those in the various time controls, along with significantly higher levels of O_2_^−^ and H_2_O_2_. Decreased cardiac cell viability was not observed in various time controls. A significant reduction in cardiac cell viability was observed in the infrasound group compared to the control, while significantly increased cardiac cell viability was observed in the infrasound exposure and rosiglitazone pretreatment group. Compared to the control, rosiglitazone significantly upregulated CAT, GPx, SOD1, and SOD2 expression and their activities in rat cardiomyocytes exposed to infrasound, while the levels of O_2_^−^ or H_2_O_2_ were significantly decreased. A potential link between a significant downregulation of PPAR-γ expression in rat cardiomyocytes in the infrasound group was compared to the control and infrasound-induced oxidative stress. These findings indicate that infrasound can induce oxidative damage in rat cardiomyocytes by inactivating PPAR-γ.

## Introduction

Recently, interest in the potential adverse health effects of infrasound (generally inaudible sound with a frequency of <20 Hz) has increased [1]. Infrasound exposure is ubiquitous in modern life, generated by natural and industrial sources. Occupational Safety and Health Administration guidelines for occupational noise exposure are concerned with sound pressure levels limits (90–115 dB for 0.25–8 h), not frequencies [2]. The majority of infrasound effects on humans appear to be annoyance [3–5]. To achieve a given level of annoyance, low frequencies were found to require greater sound pressure. In addition, small changes in sound pressure can cause significantly large changes in annoyance in the infrasonic region [5]. Infrasound exposure may also cause fatigue, headache, impaired concentration, sleep disturbance, and physiological stress. Based on the known effects of infrasound, we investigated the relationship between infrasound exposure and infrasonic bioeffects by animal experiments.

Recently [6], we reported that infrasound induced hemodynamic, cardiac ultrastructure damage and cardiac cell apoptosis in rats [6]. Furthermore, L-type calcium currents in rat ventricular myocytes were modified by infrasound exposure [7]. These experimental findings suggest that there may be important regulatory roles for infrasound on the cardiovascular system.

Due to the known effects of oxidative stress on the cardiovascular system, we speculated that infrasound exposure may induce oxidative stress in cardiac cells. PPAR-γ protects cardiomyocytes from oxidative stress in rat cardiac tissues, potentially through increased catalase expression or decreased intracellular H_2_O_2_ [8, 9]. The lack of PPAR-γ was primarily responsible for the development of intrinsic cardiac dysfunction associated with mechanical impairment of myosin [10].

Therefore, in the present study, we tested whether infrasound induced oxidative damage and whether PPAR-γ was involved in the regulation of infrasonic oxidative stress.

## Materials and Methods

### Experimental Animals and Exposure Procedures

Fifty-six neonatal male Sprague–Dawley (SD) rats (1- to 3-day-old, 12 ± 3 g) were obtained from the Center of Experimental Animal of the Fourth Military Medical University and maintained on food and water ad libitum. The rats were housed in an air-conditioned room (12/12 h light/dark cycle at a temperature of 23 ± 2 °C and a relative humidity of 60 ± 5 %). All rats were randomly divided into seven groups: four different time control groups and three experimental groups, with 24, 48, and 72 h infrasound exposure (*n* = 8 rats per group). All animal procedures were performed in accordance with institutional ethical guidelines that were approved by the animal care and use committee of the Fourth Military Medical University (Xi’an, China).

### Neonatal Rat Cardiomyocytes

Neonatal rat cardiomyocytes were prepared from the left ventricle of 1- to 3-day-old Sprague–Dawley rats as described previously [7, 11]. In order to remove fibroblasts, the cells were pre-plated and cultured at 37 °C for 90 min. Unattached cardiomyocytes were collected, counted, and seeded in 6-well culture plates at a density of 3 × 10^5^/well. Bromodeoxyuridine (0.1 mM) was added to the culture medium for the first 72 h to deplete fibroblasts, and culture medium was changed every 48 h. Cardiomyocytes were randomly divided into seven groups: 0-h control group, 24-h control group, 48-h control group, 72-h control group, 24-h infrasound exposure group, 48-h infrasound exposure group, and 72-h infrasound exposure group (*n* = 8 rats per group). Cardiomyocytes were treated with infrasound at 5 Hz and 130 dB and/or rosiglitazone at various concentrations for 0, 24, 48, and 72 h. As previously reported by Samuel Bell D et al. [12], the concentrations of rosglitazone were effective in these experiments.

### Western Immunoblotting

For the preparation of whole-cell lysates, cultured cells were washed with ice-cold PBS and lysed for 30 min on ice in RIPA buffer containing 150 mM NaCl as described [13]. Cell lysates were cleared at 20,000 g for 10 min. Following adjustment for protein concentration (Bradford assay), the lysates were boiled in SDS sample-loading buffer for 5 min and then separated using SDS–polyacrylamide gel electrophoresis (PAGE, 4–15 %, Bio-Rad). Gels were blotted on a polyvinylidene difluoride membrane (Immobilon P; Millipore, Bedford, MA, USA) and stained with the indicated first antibody purchased from Santa Cruz Biotechnology Inc. (Santa Cruz, CA) or Calbiohem (Cambridge, MA, USA). Antibody binding was detected using horseradish peroxidase-coupled secondary antibody followed by chemiluminescence detection (ECL Plus; Amersham Pharmacia, Uppsala, Sweden). The relative amounts of protein expression were calculated using the expression of rat alpha-actin (actin-α) as an internal standard.

### RNA Extraction and Reverse Transcriptase PCR

Total RNA was isolated using TRIzol reagent (Invitrogen). From each cardiac sample, 1 μg of RNA was reverse transcribed using random primers, and AMV reverse transcriptase was performed according to the manufacturer’s protocol (Promega). Polymerase chain reaction (PCR) was performed in a Mastercycler (Eppendorf, Germany) with the primers indicated in Table 1. Conditions for PCR were 1 × (94 °C C for 4 min); 35 × (94 °C for 45 s; 58 °C for 42 s; and 72 °C for 1 min); and 1 × (72 °C for 10 min). Amplification products obtained by PCR were electrophoretically separated using a 1 % agarose gel and visualized by ethidium bromide (EtBr) staining. The relative amounts of gene expression were calculated using the expression of actin-α as an internal standard.

**Table 1:** Primers used in RT-PCR

Target genes	Sequence of primers
CAT	Sense 5′-ATGGCTTTTGACCCAAGCAA-3′
	Antisense 5′-CGGCCCTGAAGCTTTTTGT-3′
GPx	Sense 5′-GCGGGCCCTGGCATTG-3′
	Antisense 5′-GGACCAGCGCCCATCTG-3′
SOD1	Sense 5′-CACTCTAAGAAACATGGCG-3′
	Antisense 5′-CTGAGAGTGAGATCACACG-3′
SOD2	Sense 5′-TTCAGCCTGCACTGAAG-3′
	Antisense 5′-GTCACGCTTGATAGCCTC-3′
PPAR-γ	Sense 5′-ACT GCC TAT GAG CAC TTC AC-3′
	Antisense 5′-CAA TCG GAT GGT TCT TCG GA-3′
Actin-α	Sense 5′-TCCCTGTACGCTTCTGGTCGTA-3′
	Antisense 5′-TCTCAAAGTCCAAAGCCACATA-3′

### Measurement of Catalase (CAT) Activity

As previously reported by Chan et al. [14], the activity of CAT from cultured cardiomyocytes was measured using the Amplex Red CAT Assay Kit (MolecularProbes, Eugene, OR, USA). In brief, reaction mixtures contained 50 μM Amplex red reagent, 40 μM H_2_O_2_, 0.2 units/ml horseradish peroxidase, and total protein (1 mg protein/ml), and then, they were incubated at room temperature for 30 min. CAT activity was determined by measuring the absorbance at 570 nm using a Bio-Radmicroplate reader. CAT activity (U) was calculated as the amount of sample required to hydrolyze 1 μmol of H_2_O_2_ per minute, based on the molecular absorbance of 0.04 × 10^6^ for H_2_O_2_.

### Measurement of Glutathione Peroxidase (GPx) Activity

As previously reported by Chan et al. [14], the activity of GPx was measured using a GPx Cellular Activity Assay Kit (Sigma). Cultured cardiomyocytes were homogenized in 0.1 M PBS and centrifuged at 13,000 g (4 °C) for 15 min. Fifty microliters of the supernatant was added to 950 μl of 50 mM Tris–HCl buffer containing 0.5 mM EDTA, 5 mM NADPH, 42 mM reduced glutathione, and 10 units/ml glutathione reductase. The reaction was initiated by the addition of 30 mM t-butyl hydroperoxide, and oxidation of NADPH was detected by monitoring the decrease in absorbance at 340 nm. GPx activity is expressed as U/mg protein. One unit of GPx activity was defined as the amount of sample required to oxidize 1 μmol of NADPH per minute, which was based on the molecular absorbance of 6.22 × 10 ^6^ for NADPH.

### Measurement of Superoxide Dismutase (SOD) Activity

The activity of SOD in cultured cardiomyocytes was measured using an SOD assay kit (Calbiochem, SanDiego, CA, USA). The assay kit utilized 5, 6, 6a, 11b-tetrahydro-3,9,10-trihydroxybenso fluorine as the substrate. This reagent undergoes alkaline autoxidation, which is accelerated by SOD, and yields a chromophore that absorbs maximally at 525 nm. The respective activities of SOD1 or SOD2 in rat cultured cardiomyocytes were measured according to the manufacturer’s instructions. A 50 % inhibition was defined as 1 unit of SOD, and the specific activity is expressed as U/mg protein of rat cultured cardiomyocytes [15].

### Superoxide Anion(O_2_^−)^ Detection

O_2_^−^ production was determined using lucigenin-enhanced chemiluminescence according to previously described and validated methods [12, 16, 17]. Briefly, cultured cardiomyocytes were homogenized in a 20 mM sodium phosphate buffer (pH 7.4), contained 0.01 mM EDTA, by a glass-to-glass homogenizer. The homogenate was subjected to low-speed centrifugation at 1,000 g for 10 min at 4 °C to remove nuclei and unbroken cellular debris. The pellet was discarded and the supernatant was obtained immediately for O_2_^−^ measurement. Background chemiluminescence in a buffer (2 ml) that contained lucigenin (5 μM) was measured for 5 min. An aliquot of 100 μl of the supernatant was then added, and chemiluminescence was measured for 10 min at room temperature (Sirius Luminometer, Berthold, Germany). O_2_^−^ production was calculated and expressed as μmol/min/mg protein. Specificity for O_2_^−^ was determined by the addition of SOD (350 U/ml) into the incubation medium.

### Hydrogen Peroxide (H_2_O_2_) Detection

H_2_O_2_ production in cultured cardiomyocytes was assessed using the Amplex Red Hydrogen Peroxide/Peroxidase Assay Kit (Molecular Probes) [18, 19]. Reaction mixtures contained 50 μM Amplex red reagent, 0.1 units/ml peroxidase, and cultured cardiomyocytes (1 mg protein/ml) that were then incubated at room temperature for 30 min [18]. H_2_O_2_ levels were determined by measuring the absorbance at 570 nm and expressed as pmol/min/mg protein using a standard curve.

### Cell Viability Assay

Cardiac cells were seeded in 24-well plates and cultured as mentioned above. 3,4,5-Dimethylthiazol-2-yl-2,5-diphenyltetrazolium (MTT) (Sigma) was dissolved in PBS containing 0.5 g/l glucose and 10 mg/l CaCl_2_ at a concentration of 1 mg/ml. Cells were washed twice with PBS and then incubated with the MTT solution for 1.5 h at 37 °C. Cells were then lysed with propan-2-ol and HCl (0.1 N), and then, the medium was transferred into 96-well plates. Absorbance of the reaction solution at 620 nm was measured using an ELISA plate reader (Titertek Multiskan MCC/340).

### Statistical Analyses

Data are expressed as mean ± SEM. Statistical analyses were performed using SPSS, version 13.0 (SPSS Science, Chicago, IL, USA). *P* < 0.05 was considered significant. The differences between the means were determined using one-way analysis of variance (ANOVA). If significant, group means were compared using least significant difference methods for multiple comparisons of means.

## Results

Infrasound Exposure Induced Oxidative Stress in Rat Cardiomyocytes.

**Fig. 1:**
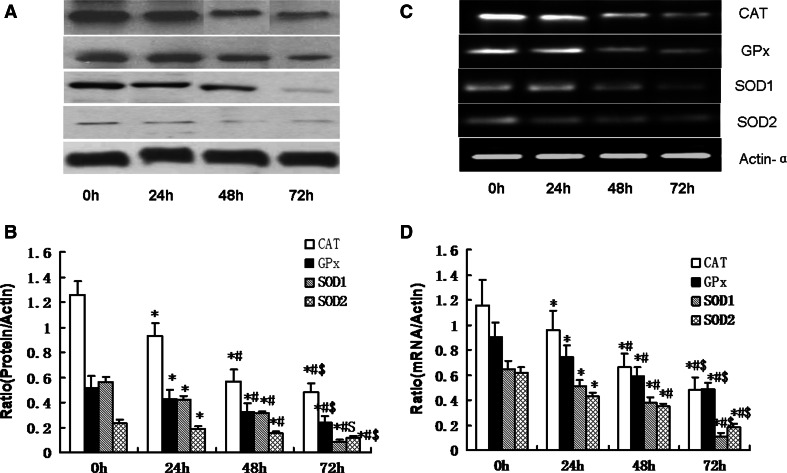
The expression of CAT, GPx, SOD1, and SOD2 in cultured rat myocytes which were exposed to infrasound of 5 Hz at 130 dB for 0, 24, 48, and 72 h, respectively, with untreated myocytes as control. **a** Representative immunoblots. **b** Quantitation of CAT, GPx, SOD1, and SOD2 relative protein levels, the intensities of CAT, GPx, SOD1, and SOD2 products were normalized to those of human actin-α products as ratios to produce arbitrary units. **c** RT-PCR results of CAT, GPx, SOD1, and SOD2 mRNA. **d** Relative expression of CAT, GPx, SOD1, and SOD2 mRNA normalized with the internal marker human actin-α. The statistical significance: *p < 0.01 versus control (0 h); ^#^ *p* < 0.01 versus 24 h group; ^$^ *p* < 0.01 versus 48 h group. Mean ± SE (*n* = 8)

**Fig. 2:**
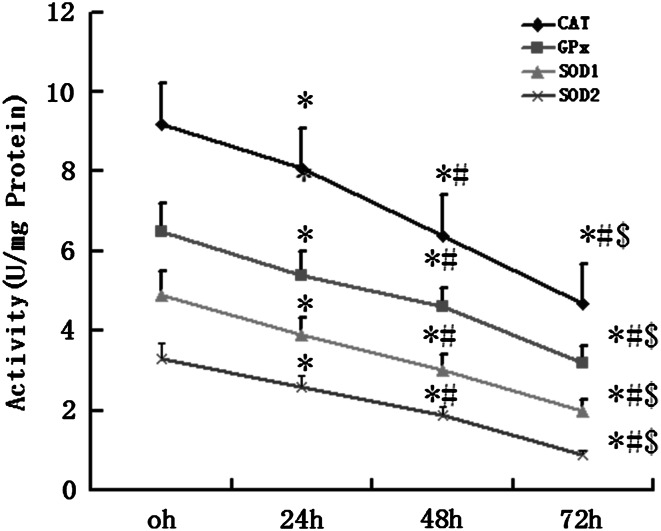
Measurement of enzyme activities of CAT, GPx, SOD1, and SOD2 in the myocytes which were exposed to infrasound of 5 Hz at 130 dB for 0 h, 24 h, 48 h, and 72 h, respectively, with untreated myocytes as control. The statistical significance: *p < 0.01 versus control (0 h); ^#^ *p* < 0.01 versus 24 h group; ^$^ *p* < 0.01 versus 48 h group. Mean ± SE (*n* = 8)

**Fig. 3:**
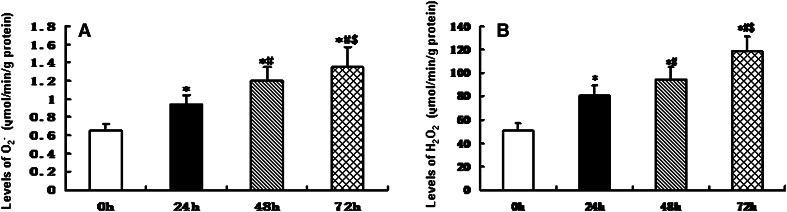
Levels of O_2_^−^ and H_2_O_2_ detected in rat myocytes which were exposed to infrasound of 5 Hz at 130 dB for 0, 24, 48, and 72 h, respectively, with untreated myocytes as control. **a** The effect of infrasound on the levels of O_2_^−^ in cultured rat myocytes. **b** The effect of infrasound on the levels of H_2_O_2_ in cultured rat myocytes. The statistical significance: *p < 0.01 versus control (0 h); ^#^ *p* < 0.01 versus 24 h group; ^$^ *p* < 0.01 versus 48 h group. Mean ± SE (*n* = 8)

In order to determine whether infrasound was associated with cardiac oxidative damage, rats were exposed to infrasound at 5 Hz and 130 dB for 0, 24, 48, and 72 h, respectively. Untreated rats served as time controls. As shown in Fig. 1, the expression of CAT, GPx, SOD1, and SOD2 was significantly decreased in the cardiac cells in the infrasonic exposure group in a time-dependent manner. As shown in Fig. 2, the enzyme activities of CAT, GPx, SOD1, and SOD2 were significantly decreased in the cardiomyocytes from the infrasonic exposure group in a time-dependent manner. Compared with time controls, the levels of O_2_^−^ or H_2_O_2_ were significantly higher in the cardiomyocytes from the infrasonic exposure group, as compared to controls (Fig. 3).

### Infrasound Exposure Decreased PPAR-γ Expression in Cultured Rat Cardiomyocytes

**Fig. 4:**
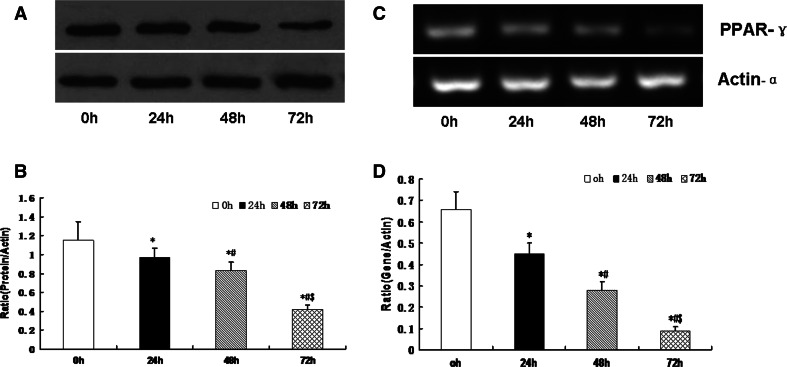
The expression of PPRA-γ in cultured rat myocytes was exposed to infrasound of 5 Hz at 130 dB for 24, 48, and 72 h, respectively, with untreated myocytes as control. **a** Representative immunoblots of PPRA-γ in cultured cardiac cells. **b** Quantitation of PPRA-γ relative protein levels in cultured cardiac cells, the intensities of PPRA-γ products were normalized to those of human actin-α products as ratios to produce arbitrary units. (C) RT-PCR results of PPRA-γ mRNA cultured cardiac cells. (D) Relative expression of PPRA-γ mRNA normalized with the internal marker actin-α. The statistical significance: *p < 0.01 versus control (0 h); ^#^ *p* < 0.01 versus 24 h group; ^$^ *p* < 0.01 versus 48 h group. Mean ± SE (n = 8)

It has been reported that declined expression of PPAR-γ protein in heart cells is associated with an increase in cardiac oxidative stress [17]. We examined the involvement of PPAR-γ in cultured cardiomyocytes exposed to infrasound. Compared with the time controls, PPAR-γ expression was significantly decreased in the infrasound-exposed group (Fig. 4).

### The PPAR-γ Agonist Rosiglitazone Attenuated Infrasound-Induced Cytotoxicity

**Fig. 5:**
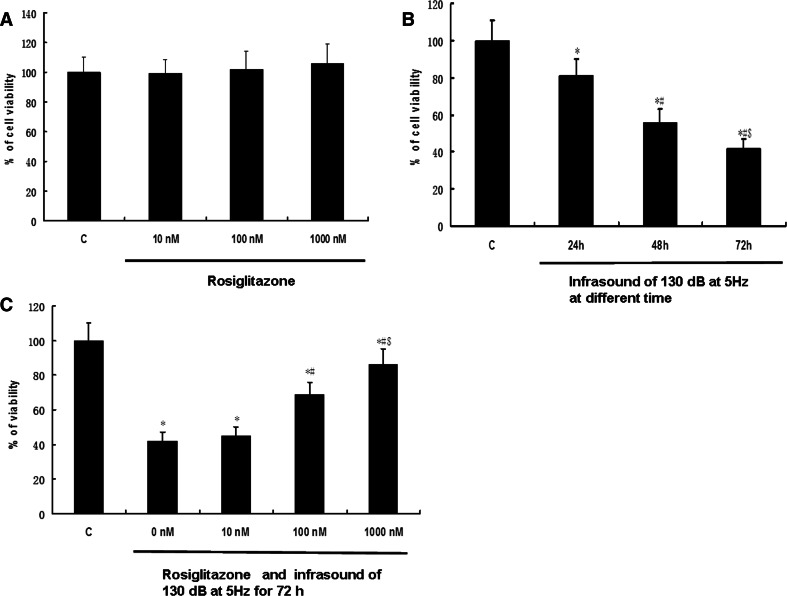
The changes of cell viability of cultured rat myocytes exposed to infrasound of 5 Hz at 130 dB for 24, 48, and 72 h, respectively, with untreated myocytes as control. **a** The changes of cell viability of cultured rat myocytes in rosiglitazone (from 10 to 1,000 nM) pretreatment groups. **b** The changes of cell viability of cultured rat myocytes in infrasound exposure groups from 24 to 72 h. (I). The statistical significance: *p < 0.01 versus control (0 h); #*p* < 0.01 versus 24 h group; $*p* < 0.01 versus 48 h group. **c** The changes of cell viability of cultured rat myocytes in infrasound exposure (for 72 h) and rosiglitazone pretreatment (from 10 to 1,000 nM) group (I + R). The statistical significance: *p < 0.01 versus control; #*p* < 0.01 versus rosiglitazone of 10 Mn group; $*P* < 0.01 versus rosiglitazone of 100 Mn group. Mean ± SE (*n* = 8)

Using the MTT cytotoxicity test, we examined whether the cytoprotective effects of rosiglitazone prevented infrasound-induced oxidative stress. We first pretreated the cells with concentrations of rosiglitazone ranging from 10 to 1,000 nM. Compared to the different controls, the reduction in cell viability was not observed in a concentration-dependent manner in the different concentration rosiglitazone pretreatment groups (Fig. 5a). We then determined the cytotoxicity of infrasound at 5 Hz and 130 dB from 24 h to 72 h. A significant decrease in cell viability was observed (*p* < 0.01) in a time-dependent manner in the infrasound exposure group (Fig. 5b) compared to control. As shown in Fig. 5c, when the cells were pretreated with rosiglitazone (from 100 to 1,000 nM), prior to infrasound-induced oxidative stress for 72 h, there were a significant decrease in cell viability (*p* < 0.01) in cardiac cells pretreated with rosiglitazone (0 nM) in the infrasound group compared to control, no change in cell viability in cardiac cells pretreated with rosiglitazone (10 nM) and a significant increase in cell viability (*p* < 0.01) in cardiac cells pretreated with rosiglitazone (from 100 to 1,000 nM) in a concentration-dependent manner in the infrasound and rosiglitazone pretreatment groups compared to the infrasound exposure group.

### Rosiglitazone Inhibited Infrasound-Induced Oxidative Stress

**Fig. 6:**
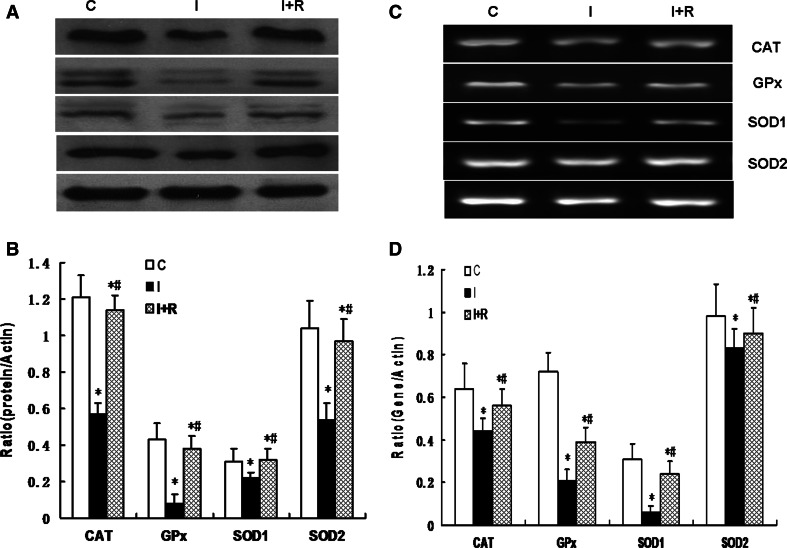
The effect of rosiglitazone on the expression of CAT, GPx, SOD1, and SOD2 in cultured rat myocytes exposed to infrasound of 5 Hz at 130 dB for 72 h. Cultured rat myocytes were randomized into control group with untreated cells (C), infrasound exposure group (I) and infrasound exposure and rosiglitazone (1,000 nM) pretreatment group (I + R), respectively. **a** Representative immunoblots. **b** Quantitation of CAT, GPx, SOD1, and SOD2 relative protein levels, the intensities of CAT, GPx, SOD1, and SOD2 products were normalized to those of human actin-α products as ratios to produce arbitrary units. (C) RT-PCR results of CAT, GPx, SOD1, and SOD2 mRNA. (D) Relative expression of CAT, GPx, SOD1, and SOD2 mRNA normalized with the internal marker human actin-α. The statistical significance: *p < 0.01 versus control; ^#^ *p* < 0.01 versus infrasound exposure group. Mean ± SE (*n* = 8)

**Fig. 7:**
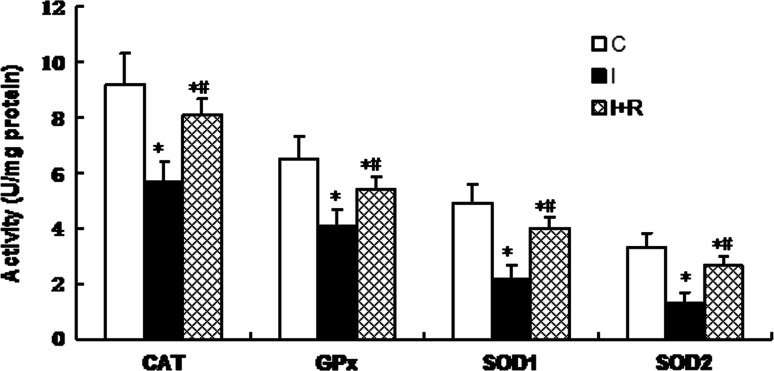
The effect of rosiglitazone on the activities of CAT, GPx, SOD1, and SOD2 in cultured rat myocytes exposed to infrasound of 5 Hz at 130 dB for 72 h. Cultured rat myocytes were randomized into control group with untreated cells (C), infrasound exposure group (I) and infrasound exposure and rosiglitazone (1,000 nM) pretreatment group (I + R), respectively. The statistical significance: *p < 0.01 versus control; ^#^ *p* < 0.01 versus infrasound exposure group. Mean ± SE (*n* = 8)

**Fig. 8:**
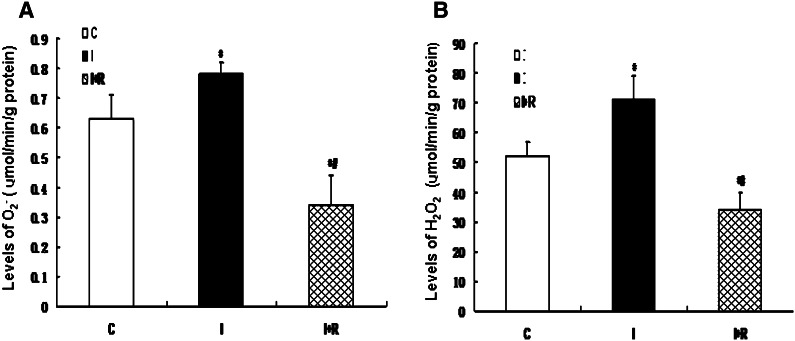
The effect of rosiglitazone on the levels of O_2_^−^ and H_2_O_2_ in cultured rat myocytes exposed to infrasound of 5 Hz at 130 dB for 72 h. Cultured rat myocytes were randomized into control group with untreated cells (C), infrasound exposure group (I), and infrasound exposure and rosiglitazone (1,000 nM) pretreatment group (I + R), respectively. **a** The effect of rosiglitazone on the levels of O_2_^−^ in cultured rat myocytes. **b** The effect of rosiglitazone on the levels of H_2_O_2_ in cultured rat myocytes. The statistical significance: *p < 0.01 versus control; ^#^ *p* < 0.01 versus infrasound exposure group. Mean ± SE (*n* = 8)

Cultured rat cardiomyocytes were randomized into a control group with untreated cells (C), an infrasound exposure group (I), and an infrasound exposure and rosiglitazone (1,000 nM) pretreatment group (I + R), respectively. Cardiomyocytes were exposed to infrasound at 130 dB and 5 Hz for 72 h in the infrasound exposure group. As shown in Fig. 6, the expression of CAT, GPx, SOD1, and SOD2 was significantly decreased in the infrasound exposure group and in the infrasound exposure and rosiglitazone pretreatment group compared to control (*p* < 0.01) and significantly increased (*p* < 0.01) in the infrasound exposure and rosiglitazone pretreatment group compared to the infrasound exposure group. As shown in Fig. 7, the enzyme activity of CAT, GPx, SOD1, and SOD2 was significantly decreased (*p* < 0.01) in the infrasound exposure group and in the infrasound exposure and rosiglitazone pretreatment group compared to control and significantly increased (*p* < 0.01) in the infrasound exposure and rosiglitazone treatment group compared to the infrasound exposure group. In addition, as shown in Fig. 8, the levels of O_2_^−^ or H_2_O_2_ were significantly increased (*p* < 0.01) in the infrasound exposure group compared to the control and significantly decreased (*p* < 0.01) in the infrasound exposure and rosiglitazone pretreatment group compared to the control and the infrasound exposure group.

## Discussion

High pressure levels of infrasound may induce resonance responses in body cavities [1, 20]. Man-made infrasonic sources and the potential harm of infrasound are increasing. Infrasound causes biological resonance, which can directly and indirectly induce a series of bioeffects. Some infrasound exposures result in death of the whole organism, organs, tissues, and cells [20]. In this study, we investigated the action of infrasound and analyzed its underlying mechanisms during the course of oxidative damage.

The present study provides novel evidence to suggest that a reduction in gene expression, protein expression, and antioxidant enzyme activities of CAT, GPx, SOD1, and SOD2 is associated with an increase in O_2_^−^ or H_2_O_2_ levels in cultured rat cardiomyocytes [21–23]. We also demonstrated that these biochemical correlates of oxidative stress are related to oxidative damage by reduced expression or decreased activities of antioxidant enzymes and augmented oxidant O_2_^−^ or H_2_O_2_ levels in cultured cardiomyocytes.

Our experimental findings revealed that gene expression, protein expression, and antioxidant enzyme activities of SOD1, SOD2, CAT, and GPx were significantly reduced in cultured cardiomyocytes exposed to infrasound (Figs. 1 and 2). This decrease in gene expression, protein expression, and antioxidant enzyme activities of SOD1, SOD2, CAT, and GPx was accompanied by a significantly augmented oxidative stress, as exemplified by an increased level of O_2_^−^ or H_2_O_2_ in cultured rat cardiomyocytes from exposed animals (Fig. 3). In fact, it has been reported that GP_X_ mRNA levels are increased in skeletal muscle cells treated with H_2_O_2_ [19]. Elimination of H_2_O_2_ is critical to protect heart tissue from oxidative stress. Superoxide is converted to H_2_O_2_ by SOD in the presence of myocytes, while H_2_O_2_ forms one of the most toxic oxygen free radicals. In the myocardium, GPx plays an important role in the scavenging of H_2_O_2_, while CAT, the other major H_2_O_2_ scavenging enzyme, has very low activity [21]. The actions of SOD1, SOD2, and CAT are similar to those of GPx in the course of infrasonic oxidative damage.

PPARs are members of the nuclear receptor superfamily. Cardiomyocytes express all three PPAR subtypes (α, β/δ, and γ). It is now clear that PPAR-γ plays a critical role in myocardial fatty acid oxidation [24]. In this study, oxidative stress appeared to be linked to profound alterations in SOD expression and activity. As a potential link between the downregulation of the PPAR-γ pathway and the progression of cardiac dysfunction, functional PPAR-γ is required to regulate cellular oxidant–antioxidant balance, prevent oxidative damage, and preserve contractile function in cardiac muscle [21–23]. In our study, the expression levels of PPAR-γ were all significantly decreased in myocytes from infrasound-exposed animals in a time-dependent manner (Fig. 4), which may be responsible for infrasonic oxidative damage.

Rosiglitazone is a PPAR-γ selective agonist. In our experiment, PPAR-γ inactivation was observed in the infrasound exposure group. Infrasound induced a significant decrease in cell viability, as measured by the MTT test, significantly higher levels of O_2_^−^ and H_2_O_2_, and significantly decreased mRNA and protein expression levels of CAT, GPx, SOD1 and SOD2. Compared to the cell viability of the infrasound exposure group, cell viability was significantly increased in the infrasound and rosiglitazone pretreatment group, along with significantly decreased levels of O_2_^−^ and H_2_O_2_ and significantly increased mRNA and protein expression levels of CAT, GPx, SOD1 and SOD2. Our study demonstrates that PPAR-γ is involved in the regulation of infrasonic oxidative stress. Cells pretreated with the PPAR-γ selective agonist rosiglitazone inhibited infrasound-induced oxidative stress. Indeed, PPAR-γ activation led to inhibition of H_2_O_2_ and O_2_^−^ and upregulated the expression of the antioxidants CAT, GPx, SOD1, and SOD2. Therefore, rosiglitazone may protect from infrasound-induced oxidative stress.

Previously, Zhaohui Pei et al. [1, 20] reported that infrasound affected the apoptotic rates of rat cardiomyocytes. This suggests that the apoptotic-inducing effects of infrasound may be cell-specific, and cardiomyocytes might be more sensitive to infrasound than other types of cells [1]. Rats exposed to 8 or 16 Hz at 120–140 dB for up to 40 days showed reduced oxidation–reduction (redox) enzymes in the myocardium, disturbed blood flow, myofibrillar fragmentation, and RNA and DNA alterations [14, 25]. Thus, it is important to protect the heart from infrasound damage. In addition, rats acutely exposed to infrasonic oscillations for 15, 30, and 60 min showed frequency-dependent phasic changes in cholinergic activity, as determined by measurements of acetylcholine and acetylcholinesterase [26].

In summary, acute infrasound exposure induces oxidative damage of cardiomyocytes that affects a series of oxidative damage-related proteins and genes, suggesting a complex signaling network that is evoked by infrasound. PPAR-γ is also involved in infrasonic cardiac oxidant–antioxidant balance.
